# Targeting Immune Signaling Checkpoints in Acute Myeloid Leukemia

**DOI:** 10.3390/jcm8020236

**Published:** 2019-02-12

**Authors:** Krzysztof Giannopoulos

**Affiliations:** 1 Department of Experimental Hematooncology, Medical University of Lublin, 20-093 Lublin, Poland; krzysztof.giannopoulos@gmail.com or krzysztof.giannopoulos@umlub.pl; Tel.: +48-81-448-66-32; Fax: +48-81-448-66-34; 2 Department of Hematology, St John’s Cancer Centre, 20-093 Lublin, Poland

**Keywords:** acute myeloid leukemia, immunotherapy, programmed cell death protein 1 receptor/ programmed death-ligand 1 (PD-1/PD-L1) signaling

## Abstract

The modest successes of targeted therapies along with the curative effects of allogeneic hematopoietic stem cell transplantation (alloHSCT) in acute myeloid leukemia (AML) stimulate the development of new immunotherapies. One of the promising methods of immunotherapy is the activation of immune response by the targeting of negative control checkpoints. The two best-known inhibitory immune checkpoints are cytotoxic T-lymphocyte antigen-4 (CTLA-4) and the programmed cell death protein 1 receptor (PD-1). In AML, PD-1 expression is observed in T-cell subpopulations, including T regulatory lymphocytes. Increased PD-1 expression on CD8+ T lymphocytes may be one of the factors leading to dysfunction of cytotoxic T cells and inhibition of the immune response during the progressive course of AML. Upregulation of checkpoint molecules was observed after alloHSCT and therapy with hypomethylating agents, pointing to a potential clinical application in these settings. Encouraging results from recent clinical trials (a response rate above 50% in a relapsed setting) justify further clinical use. The most common clinical trials employ two PD-1 inhibitors (nivolumab and pembrolizumab) and two anti-PD-L1 (programmed death-ligand 1) monoclonal antibodies (atezolizumab and durvalumab). Several other inhibitors are under development or in early phases of clinical trials. The results of these clinical trials are awaited with great interest in, as they may allow for the established use of checkpoint inhibitors in the treatment of AML.

## 1. Introduction

The modest successes of targeted therapies in acute myeloid leukemia (AML) and the proven power of the immune system to fight effectively against leukemic blasts in the context of the graft-versus-leukemia effect, stimulate the development of new immunotherapies. One of the promising methods of immunotherapy is the activation of immune response by the targeting of negative control checkpoints on the surface of immune cells or by eliminating the regulatory proteins present in the tumor microenvironment [1]. The homeostasis of the stimulating and inhibiting signals of the immune response is regulated by immunological control checkpoints, which allow the activation of cytotoxic response against pathogens, while maintaining a tolerance to the organism’s own cells.

**Figure 1:**
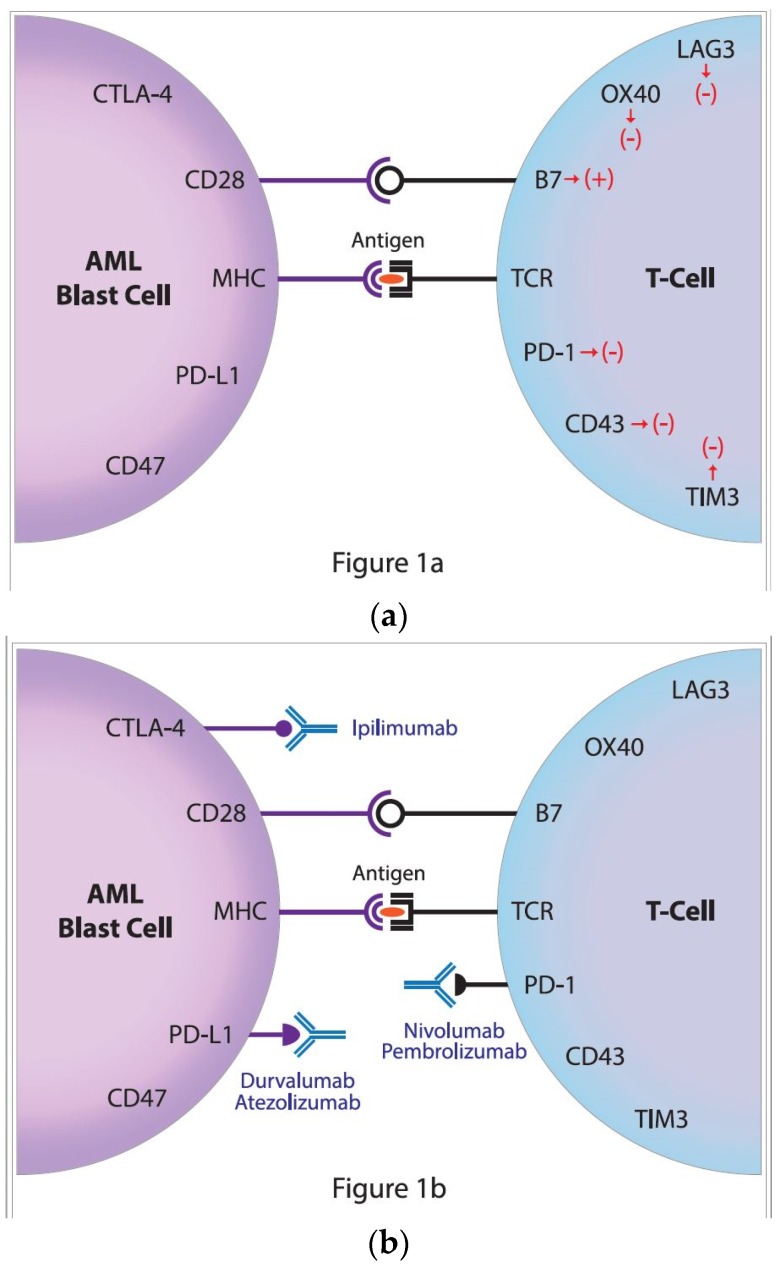
(**a**) The regulation of antileukemic immune response; the activation of an immune response requires two signals. The first is responsible for the specific recognition of antigen (peptide) located in the great groove of the major histocompatibility complex (MHC) by the T-cell receptor (TCR). The second is the costimulatory signal that is transmitted by the interaction of CD28 and B7 molecules. These signals are regulated by many negative receptors including the lymphocyte-activation gene 3 (LAG3), OX40, programmed cell death protein 1 receptor (PD-1), CD43 as well as T-cell immunoglobulin domain and mucin domain 3 (TIM3) on the T cell that interacts with ligands on acute myeloid leukemia (AML) blasts, i.e., CD47, programmed death-ligand 1 (PD-L1) and Cytotoxic T-lymphocyte Antigen-4 (*CTLA*-*4*). (**b**) The restoration of antileukemic immune response by the targeting of the negative control checkpoints. In order to restore the antileukemic immune response, two inhibitory immune checkpoint molecules might be targeted by treatment with specific monoclonal antibodies directed against the cytotoxic T-lymphocyte antigen-4 (CTLA-4), ipilimumab, and the programmed cell death protein 1, nivolumab and pembrolizumab as well as PD-L1, atezolizumab and durvalumab.

To avoid autoimmunity, the process of T-cell activation must be strictly regulated. For stimulation, T cells require at least two different signals from antigen presenting cells (APC). The first signal is the recognition of the antigen, and more specifically its immunogenic part—an epitope presented by the major histocompatibility complex (MHC), located on the APC and recognized by the T-cell receptor (TCR) (Figure 1a). The second signal is the result of costimulation, mainly by the interaction of the CD28 molecule of the T-lymphocyte with CD80 (B7.1) or CD86 (B7.2) molecules, on the APC. The stimulation of T cells is also closely related to other stimulatory signals sent to the cell as a result of the combination of specific receptor pairs and their ligands, including the glucocorticoid-induced TNF (tumor necrosis factor) receptor and its specific ligand, and also the interaction between the transmembrane 4-1BB receptor (CD137) and its ligand, 4-1BBL (CD137L) [2,3,4].

The two best-known inhibitory immune checkpoints are cytotoxic T-lymphocyte antigen-4 (CTLA-4) and the programmed cell death protein 1 receptor(PD-1) (Figure 1a) [1]. On T cells, the CTLA-4 receptor inhibits T cell maturation and differentiation by competing with the costimulatory receptor CD28, for CD80 (B7.1) and CD86 (B7.2) [5]. Although the increased CD80 and CD86 expressions that are associated with poor outcome were reported in AML, treatment with the anti-CTLA-4 monoclonal antibody ipilimumab, proved limited clinical activity [6,7,8]. Encouraging results from trials on solid tumors have turned the attention of researchers to the potential that blocking the PD-1 signaling pathway may have potential applications in the field of hematology–oncology. Those new therapies might target the immune synapse of patients irrespective of the PD-1 expression and thereby could be proposed for a majority of AML patients (Figure 1b).

## 2. The Role of PD-1/PD-L1 Signaling Pathway

PD-1 is a surface glycoprotein cell receptor that belongs to the CD28 family. PD-1 is composed of 288 amino acids, and its molecular weight ranges from 50 to 55 kDa2. PD-1 exhibits approximately 31–33% homology with CTLA-4, CD28, and Inducible T-cell COStimulator (ICOS) molecules. PD-1 interactions with ligands prevent autoimmunity on the one hand, by inducing apoptosis of autoantigen-specific T cells and on the other hand, by inhibiting regulatory T cell (Treg) apoptosis. The expressions of PD-1 on T and B cells is a consequence of the activation of the signaling pathway TCR or the B-cell receptor (BCR), respectively [9].

The PD-1 protein is encoded by the *PDCD-1* gene, located on chromosome 2 (2q.37.3) [10]. *PDCD-1* consists of five exons. Exon 1 encodes a leader peptide that is extracellular. Exon 2 encodes the immunoglobulin (Ig) variable (V-like domain. Amino acid fragments (ca. 20) are located at the IgV-like domain, that separates it from the cell membrane. A transmembrane domain encapsulated by exon 3 is anchored within the cell membrane. Exons 4 and 5 encode an intracellular domain, in which we distinguish two tyrosines, located in two amino acid motifs—proximal (tyrosine-based motif inhibitors—ITIM) and distal (a tyrosine immunoreceptor-based switch motif—ITSM) [11]. The tyrosines mentioned above play a fundamental role in the function of PD-1 as an inhibitor [12]. Under physiological conditions, PD-1 is expressed on the cells of the immune system, including mature CD4+ and CD8+ T cells, as well as on B cells and T cells during their thymus development [13,14]. In addition, PD-1 expression is found on natural killer (NK) cells, some dendritic cell (DC) subpopulations, and monocytes [15,16]. In a form unrelated to the cell membrane, PD-1 may be present in the cytoplasm of Treg and naїve CD4+ cells. PD-1 can be regulated by various factors, including hormones, cytokines or suppressor genes, such as Phosphatase and tensin homolog (*PTEN)* and liver kinase B1 (*LKB1*) [17]. The cytokines that stimulate the expression of PD-1 are interleukin 2 (IL-2), IL-7, IL-15 and IL-21. It has been shown that in the induction of PD-1 expression in T cells, there is a significant role played by the nuclear factor of stimulated Tc1 cells (NF-ATc1). It has also been proven that the specific inhibition of this factor, consisting in the abolition of its translocation to the nucleus, results in the reduction of PD-1 expression, and the mutation of the gene encoding NF-ATc1 results in the complete lack of receptor expression [18]. The transmission of the signal through TCR after its stimulation leads to the binding of NFAT to the promoter region of the *PDCD1* gene [18]. PD-1 expression in B-lymphocytes is induced by the molecules that stimulate the activation and the proliferation of these lymphocytes, including anti-IgM, anti-CD40 and lipopolysaccharide (LPS) [9]. The interaction with toll-like receptors (TLRs) such as TLR2, TLR3, TLR4 and the nucleotide-binding oligomerization domain (NOD) has a stimulating effect on the expression of PD-1 in DC. In turn, IL-4 and TLR9 act to inhibit the expression of PD-1 in DC [19]. In macrophages, PD-1 expression is stimulated by an interferon-stimulated response element (ISRE), signal transducers and activators of transcription (STAT), including STAT1 and STAT2, and interferon α (IFNα), through ISRE [20].

The programmed death-ligand 1 (PD-L1), also referred to as B7-H1 or CD274, is a transmembrane type I glycoprotein, made up of 290 amino acids, belonging to the B7 family. This protein has two extracellular IgV- and Ig constant (C)-like domains, wherein the IgV-like domain allows for interaction with the analogous domain of the PD-1 receptor. The cytoplasmic domain of the PD-L1 ligand is short, and its exact role in the transmission of intracellular signals has not yet been determined [21]. The expression of PD-L1 at the mRNA level is detected in almost all cells. The expression of the PD-L1 protein on hematopoietic cells is limited primarily to antigen-presenting cells, such as dendritic cells, macrophages, and B28 lymphocytes. PD-L1 is also expressed in activated T cells [12]. PD-L1 is also found in tissues not belonging to the immune system, including pancreatic islet cells, hepatic stellate cells, vascular endothelial cells and placental trophoblast cells [18,22]. The expression of PD-L1 on B cells is stimulated by anti-IgM antibodies, LPS, type I and II IFNs, TNF and IL-21. In the case of T cells, the inducers of PD-L1 expression are anti-CD3 antibodies or cytokines, such as IL-2, IL-7, IL-15, IFN and TNF. The expression of PD-L1 on macrophages is stimulated by a granulocyte-macrophage-colony-stimulating factor (GM-CSF), monocytes by IL-10, and on DC by IFN-γ, IL-4, IL-12 and GM-CSF [23].

Programmed death-ligand 2 (PD-L2), also referred to as B7-DC and CD273, is the second ligand able to attach to the PD-1 receptor [24]. PD-L2 has extracellular Ig-V- and IgC-like domains, and a short intracellular domain. The expression of PD-L2, as compared with PD-L1, is not as common and is limited to macrophages, DC, and some B-cell subpopulations [25,26,27]. The partial presence of PD-L2 was also demonstrated on mast cells of myeloid origin, T-lymphocytes and vascular endothelial cells [28,29]. The PD-1 receptor interacts with its specific ligands—PD-L1 and PD-L2. Ligands compete with each other for binding to PD-1, but PD-L1 plays a major role in regulating the PD-1/PD-L1/PD-L2 pathway. Although PD-L2 has a stronger affinity for PD-1 compared to PD-L1, the extent of expression of this molecule is limited [30]. The expression of PD-1 on lymphocytes may be induced by the contact of the lymphocyte receptor with an antigen [1,31]. The interaction of PD-1 with ligands results in the activation of phosphotyrosine phosphatase, containing the SH2 domain (SHP2) and the decrease in Bcl-xL expression, leading to the inhibition of phosphatidylinositol 3-kinase/serine-threonine protein kinase—(PI3K/AKT). Functionally, elevated levels of PD-1 expression are observed on tumor-infiltrating lymphocytes (TILs) that interact with tumor cells by linking to the PD-L1 and PD-L2 ligands present on them, which in turn could lead to a lymphocyte depletion. This phenomenon leads to the inhibition of the effector functions of T cells, which lose the cytotoxic ability to kill tumor cells. PD-1 also plays a significant role in T-cell adhesion, which is activated upon contact with APC. The interaction of these cells may be impaired by PD-1-derived inhibitory signals that are necessary for its interaction with PD-L1. This hypothesis is reinforced by experimental in vitro studies showing lower T-cell mobility and improved T-cell interactions with APC after blocking with PD-1 or PD-L1 antibodies [32]. Furthermore, PD-1 could inhibit T-cell adhesion and the formation of the immunological synapse [33,34].

## 3. PD-1/PD-L1 Expression in Leukemias

The expression of PD-1 in hematological malignancies has been the subject of many studies in recent years. In patients with chronic lymphocytic leukemia (CLL), PD-1 expression is observed on T lymphocytes, but also on leukemic cells [35,36,37]. In addition, we proved earlier that PD-1 expression on leukemic cells in CLL patients was higher compared to the healthy group, both at the level of the transcript and in the form of membrane protein. However, the significance of PD-1 and PD-L1 expression in the prognostic context has not been confirmed [35]. In addition, in CLL patients with an advanced disease (stage III and IV, according to the Rai classification), a higher percentage of CD4+PD-1+ T lymphocytes was observed than in patients with a less advanced disease [38].

In AML, PD-1 expression was observed in T-cell subpopulations, including CD4+ T-effector cells, Tregs and CD8+ T cells, both in untreated patients and in patients with a recurrent disease [39]. An increased PD-1 expression on CD8+ T cells may be one of the factors leading to the dysfunction of cytotoxic T cells and the inhibition of the immune response during the progressive course of AML [40]. Knaus et al. [41] characterized the T-cell exhaustion in AML at diagnosis, that diverged between responders and non-responders upon treatment. Response to therapy correlated with the upregulation of costimulatory T-cell signaling pathways, and the downregulation of inhibitory T-cell signaling pathways, indicative of the restoration of T-cell function. Notably, CD8+ T-cell dysfunction was, in part, reversible upon PD-1 blockade in vitro. By contrast, a similar expression of inhibitory molecules on T cells from patients at AML diagnosis and from age-matched healthy controls were observed [42]. However, when observed at relapse after allogeneic hematopoietic stem-cell transplantation (alloHSCT), the PD-1 expression was significantly increased, compared with its expression at diagnosis, in both CD4+ and CD8+ T cells. Notably, bone marrow CD8 T cells consisted of a higher frequency of PD-1+ cells compared with those from peripheral blood [43]. These cells were also functionally deficient, as was the case in the functional model of WT-1-specific leukemia-reactive CD8+ cells from bone marrow that released lower levels of IFN-γ, granzyme B and TNF, when compared with those from peripheral blood.

Most studies suggest that in newly diagnosed AML patients, PD-L1 expression on blasts is usually not observed [44]. However, it might depend on the detection method, since in one study the PD-L1 was expressed in 24 out of 75 AML patients [45]. The appearance of PD-L1 on AML blasts was associated with the negative course of the disease [44]. The PD-L1 overexpression in AML usually occurred during therapy, after alloHSCT and at the relapse of the disease. The PD-L1 positive rate in the relapsed/refractory group was higher than that in the de novo patient group (56.3% vs. 25.4%, *p* = 0.019). In 59 de novo patients, the complete remission (CR) rate of the PD-L1 positive group after one course of chemotherapy, was lower than that of the PD-L1 negative group (66.7% vs. 71.4%); the CR rate of PD-L1 positive group after 2 two courses of chemotherapy, was also lower than that of PD-L1 negative group (70% vs. 88.6%). The relapse rate and the proportion of refractory patients in PD-L1 positive group were higher than those in the PD-L1 negative group [45].

The factors that stimulate the expression of PD-L1 in AML were cytokines, particularly IFN-γ [46,47]. In addition, we reported that TP53 might specifically modulate the immune response to tumor antigens by regulating PD-L1 via *miR-34* and blocking its expression [48]. The *PD-L1* expression was elevated in the AML group with *TP53* mut, compared with the *TP53* wt group, with a median expression of 9.1 vs. 8.3, *p* < 0.001. In line with this finding, Goltz et al. [49], analyzing gene methylation status in AML patients, found that low PD-L1 methylation was found in the *TP53* mut group. Wang et al. [50] have shown that *PD-L1* was overexpressed in the AML samples and that the expression level was reversely correlated with *miR-34a* expression, that directly targets the 3′ untranslated region of PD-L1, thereby modulating PD-L1 expression. We also found that the highest expression of PD-L1 was in a group with a poor prognosis, compared with favorable and intermediate groups, as defined by The Cancer Genome Atlas research network’s risk stratification. The expression of PD-L1 was also associated with the number of recurrent mutations. Possibly, an increased number of driver mutations created more neoantigens, which in turn, modified the immune microenvironment and caused an increase in PD-L1 expression [51]. The expression of PD-L1 in AML is therefore associated with adverse gene mutations that affect the microenvironment of the tumor and may lead to an unfavorable clinical course of the disease [45].

## 4. Inhibition of PD-1/PD-L1 in AML

The PD-1/PD-L1/PD-L2 pathway may be inhibited by blocking the PD-1 receptor or its ligands. Blocking the PD-1 molecule itself prevents its interaction with PD-L1 and PD-L2, which is considered the most effective activation of the immune response (Figure 1b). By contrast, PD-L1 blockade affects only the PD-1/PD-L1 axis, and considering its widespread expression, the activation of the immune response might be significant. Due to the limited expression of the PD-L2 ligand, it is not suitable target for therapies that use monoclonal antibodies [52]. Thus, in actions directed toward the PD-1/PD-L1 pathway, anti-PD-1 antibodies, as well as anti-PD-L1, are used [30]. The most common clinical trials employ two PD-1 inhibitors (nivolumab and pembrolizumab) and two anti-PD-L1 monoclonal antibodies (atezolizumab and durvalumab) (Table 1). Several others are under development or are in early phases of clinical trials. The key mechanism of action in anti-PD-1 and anti-PD-L1 monoclonal antibodies, relies on blocking PD-1 interaction with PD-L1 and/or PD-L2 ligands. To minimalize the side effects mediated by the recognition of fragment crystallizable (Fc) region, atezolizumab and durvalumab have a point mutation in the Fc domain; thus, they did not induce the cytotoxicity of the antibody-dependent cellular cytotoxicity, nor the complement-dependent cytotoxicity. The upregulation of checkpoint molecules was observed after alloHSCT and therapy with hypomethylating agents, pointing to a potential clinical application in these settings [53,54]. Moreover, a higher PD-1 expression on T cells was strongly associated with leukemia relapse, post-alloHSCT [55]. This was especially the case in the subpopulation of CD8+ T cells that, characterized by the expression of two exhaustion markers, PD-1 and the T-cell immunoglobulin domain and mucin domain 3 (TIM-3), presented the strongest predictive value for leukemia relapse, post-alloHSCT. The median frequencies of CD8+ PD-1+TIM-3+ in relapsed AML were 8.6%, compared with 0.5% in patients maintaining remission. Notably, the increase of PD-1+ TIM-3+ CD8+ T cells occurred before a clinical diagnosis of leukemia relapse, suggesting their predictive value. This study might provide an early diagnostic approach and a therapeutic target for leukemia relapse, post-transplantation.

The expression of PD-1 is regulated by DNA methylation. The demethylation of the PD-1 promoter correlated with an increase in PD-1 expression. The demethylation of the PD-1 promoter correlated with a significantly worse overall response rate (8% vs. 60%, *p* = 0.014), and a trend towards a shorter overall survival (OS) (*p* = 0.11) was observed [56]. In a cohort of patients treated with hypomethylating therapy, PD-L1, PD-L2, PD-1 and CTLA4 expressions were upregulated [54]. The treatment of leukemia cells with a hypomethylating agent, decitabine, resulted in the dose-dependent upregulation of PD-L1, PD-L2, PD-1 and CTLA4. Decitabine could also increase the expression of genes involved in antigen processing and presentation by the respective promoter, demethylation. In a mouse model of colorectal cancer, a significantly larger inhibition of tumor growth and a prolongation of survival were observed after treatment with a combination of PD-1 blockade and decitabine, than in mice treated with decitabine or PD-1 blockade alone [57]. These results suggest that PD-1 signaling may be involved in resistance mechanisms to hypomethylating agents, and provide evidence that checkpoint inhibition could be a potential therapy for treating AML.

In recently published results from a phase II study, relapsed/refractory AML patients were treated with nivolumab and azacytidine [58]. The overall response rate (ORR) was 33%, including 15 (22%) patients with complete remission/complete remission with insufficient count recovery (CRi), one patient with a partial response, and seven patients with hematologic improvements (HI) that were maintained for >6 months. Six patients (9%) exhibited a stable disease for >6 months. The highest ORR (58%) was observed in hypomethylating agent-naive patients. Grades 2–4 immune-related adverse events (irAE) occurred in 16 (23%) patients. Fourteen of the 16 (88%) patients with toxicities responded to steroids, and these 14 patients were safely rechallenged with nivolumab. In this study, a total of 13% of the patients had to discontinue nivolumab (all discontinuations were due to grades 3/4 irAE; no discontinuations were due to grade 2 irAE) and were subsequently kept only on azacitidine.

The preliminary results of a phase-II study (NCT02532231) of nivolumab maintenance in high-risk AML patients, who have achieved CR following induction and consolidation chemotherapy, also showed promising results, with the rates for a 6- and a 12-month CR duration being 79% and 71%, respectively [59]. In a series of case reports, Albring et al. [60] reported three AML patients who, at relapse after alloHSCT, were treated with nivolumab. In one patient, the therapy led to CR; in another, it led to disease stabilization; a third patient failed to respond to nivolumab. The only side effects were the irAE of pancytopenia and a graft-versus-host disease of the skin in one patient, as well as muscle and joint pain in another patient.

## 5. Future Treatment Modalities with Checkpoint Inhibitors in AML

**Figure 2:**
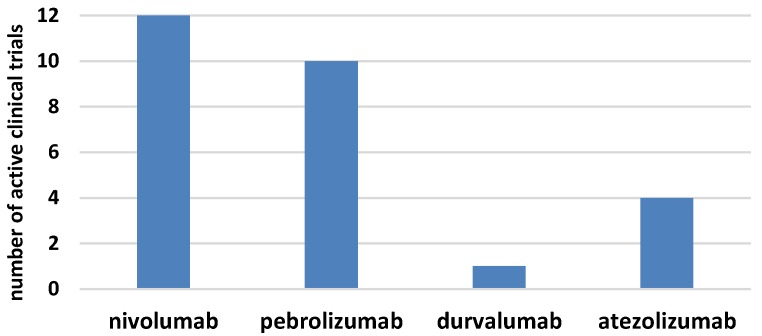
The numbers of active clinical trials for checkpoints inhibitors in acute myeloid leukemia.

The era of immunotherapy in AML started several years ago when the curative potential of alloHSCT, due to the graft-versus-leukemia effect, was discovered. Nowadays, chimeric antigen receptor (CAR)T-cell therapy replaces the defective immune system and the checkpoint inhibitors restore function to the antileukemia immune response (Figure 1b). Several phases I/II clinical trials for checkpoint inhibitors in monotherapy or combined treatment for AML have started in the last few years (Figure 2) [61,62]. The results of larger clinical trials are needed to determine the role that checkpoint inhibition plays in AML. It seems that the clinical setting can be complex, starting from combined therapy with hypomethylating drugs for patients who are ineligible for transplantation, followed by combined therapy with other modifiers of the immune system and finally, to the augmentation of the graft-versus-leukemia effect, post-alloHSCT, in either monotherapy or polytherapy. In this regard, the double blockade of CTLA-4 and PD-1/PD-L1 presents an interesting treatment option, in boosting antileukemic immunity. Recent results in solid tumors proved that in advanced melanoma, combined therapy was effective in 57% of patients, compared with 43% for patients treated with nivolumab monotherapy and 19% for patients in the ipilimumab monotherapy arm [63]. The first clinical trials on the ipilimumab and nivolumab combined treatment in patients with relapsed AML, post-alloHSCT, have been initiated. Another approach is a double blockade of TIM-3 and PD-1 that is being evaluated in an ongoing clinical trial. Furthermore, checkpoint inhibitors might be effective in combination with cellular therapies, including chimeric antigen receptor T cells (CART) or vaccination strategies [64,65]. This is an evolving field since anti-PD1/PD-L1 therapy along with vaccination could expand the pre-existing specific T cells and induce functionally active antileukemic cytotoxic T cells. The results from clinical trials in melanoma have not provided clear support for this approach, since similar clinical activity was observed irrespective of vaccination [66]. Similarly, PD-1 inhibition in combination with CART did not further enhance the expansion or persistence of CART [64]. Further results from these clinical trials and others are awaited with great interest, as they may allow for the established use of checkpoint inhibitors in the treatment of AML.

Figure 2 displays the number of active clinical trials for checkpoint inhibitors in AML both in monotherapy and in combination with chemotherapy. The histogram presents trials registered on clinicaltrial.gov [62].

## Figures and Tables

**Table 1:** The first agents for PD-1/PD-L1 inhibition in AML.

Monoclonal Antibody	Target/Type	First Registration
Nivolumab	PD-1, IgG4	Melanoma—06.2015
Pembrolizumab	PD-1, IgG4	Melanoma—08.2014
Atezolizumab	PD-L1, IgG1 modified Fc region	Urothelial carcinoma—05.2016
Durvalumab	PD-L1, IgG1 modified Fc region	Urothelial carcinoma—05.2017

PD-1: programmed cell death protein 1 receptor; PD-L1: programmed death-ligand 1; AML: acute myeloid leukemia; IgG: Intravenous Gamma Globulin.
